# A nomogram to predict survival probability of gastric cancer patients undergoing radical surgery and adjuvant chemotherapy

**DOI:** 10.3389/fonc.2022.893998

**Published:** 2022-08-05

**Authors:** Ling Ma, Guosheng Chen, Deqiang Wang, Kai Zhang, Fengjiao Zhao, Jie Tang, Jianyi Zhao, Oluf Dimitri Røe, Shaohua He, Dongcheng Liao, Yanhong Gu, Min Tao, Yongqian Shu, Wei Li, Xiaofeng Chen

**Affiliations:** ^1^ Department of Oncology, The First Affiliated Hospital of Nanjing Medical University, Nanjing, China; ^2^ Jiangsu Key Laboratory for Design and Manufacture of Micro-Nano Biomedical Instruments, Southeast University, Nanjing, China; ^3^ Pancreatic Center and Department of General Surgery, The First Affiliated Hospital of Nanjing Medical University, Nanjing, China; ^4^ Pancreas Institute of Nanjing Medical University, Nanjing, China; ^5^ The Cancer Therapy Center, Affiliated Hospital of Jiangsu University, Zhenjiang, China; ^6^ Department of Oncology, Liyang People’s Hospital, Liyang, China; ^7^ Department of General Surgery, The First Affiliated Hospital of Nanjing Medical University, Nanjing, China; ^8^ Department of Cancer Research and Molecular Medicine, Norwegian University of Science and Technology (NTNU), Trondheim, Norway; ^9^ Department of Oncology, Levanger Hospital, Nord-Trøndelag Hospital Trust, Levanger, Norway; ^10^ The Key Laboratory of Cancer Prevention and Treatment, Second People's Hospital of Huaihua City, Huaihua, China; ^11^ Department of Oncology, The First Affiliated Hospital of Soochow University, Suzhou, China

**Keywords:** gastric cancer, nomogram, prognostic scoring system, overall survival, chemotherapy

## Abstract

Gastric cancer (GC) is the third-leading cause of cancer mortality worldwide. The aim of this study was to develop a nomogram that estimates 1-year, 3-year, and 5-year survival probability of GC patients after D2 gastrectomy combined with adjuvant chemotherapy. The results showed that median age is 58 (range: 18-85) years in the training cohort and 59 (range: 32-85) years in the validation cohort. On multivariate analysis, four factors were found to be significantly associated with worse overall survival (OS): late TNM stage, positive resection margin, preoperative carcinoembryonic antigen (CEA) level, and single chemotherapy regimens compared with multiple chemotherapy regimens. All of these findings were validated in the validation cohort. Furthermore, the four factors were included in the final nomogram for the prediction of 1-year, 3-year, and 5-year survival probability, with accurate calibration and reasonable discrimination (C-index = 0.676 for training cohort, and C-index = 0.664 for validation cohort). The AUC values analyzed by the ROC analysis demonstrated a good predictive accuracy of the nomogram for OS (1-year, 3-year, and 5-year OS were 94.43%, 77.42%, and 73.03% in the training cohort, respectively; 96.95%, 81.54%, and 73.41% in the validation cohort, respectively). In conclusion, the proposed nomogram may be used to objectively and accurately predict survival probability of GC patients in a multi-institutional clinical setting.

## Introduction

Gastric cancer (GC), with an estimated mortality rate of 8.2% in 2018, is the third-leading cause of cancer mortality worldwide (1). GC is also the third most common cause of cancer-related deaths among males ranked after lung cancer and liver cancer. Radical surgery remains the only potential curative treatment in resectable GC. However, the 5-year overall survival (OS) rates of GC patients undergoing surgical resection vary a lot (2, 3). A retrospective analysis in Japan showed the 5-year OS rates of patients with surgically resected GC for pathological stage IA, IB, II, IIIA, IIIB, and IV disease as 91.5%, 83.6%, 70.6%, 53.6%, 34.8%, and 16.4%, respectively (2). In spite of novel chemotherapy regimens and targeted therapies (4), the 5-year survival for patients with advanced GC has not shown much improvements (5). Thus, further studies are required to determine better prognostic factors and treatment regimens.

Different kinds of predictive models for predicting survival probability of GC patients have been reported. Complex statistical predictive models containing large quantity of factors can be simplified to a single brief numerical estimate model *via* nomograms to predict the probability of GC patients. Previous researches have reported the predictive models of nomograms for disease-specific survival (DFS), relapse-free survival (RFS), or long-term survival after an R0 resection, or Gastrectomy with D2 lymphadenectomy (D2 gastrectomy) for GC (6–8). To predict the survival benefit from the addition of adjuvant chemotherapy for patients with stage II or stage III GC, Jiang et al. developed a survival prediction model using a nomogram (9). However, few nomograms are available for all the factors concerning patient characteristics, tumor characteristics, preoperative serum markers, and chemotherapy regimens. In addition, D2 gastrectomy is the recommended surgical approach for patients with resectable GC in Europe, United States and East Asia (10–13). Therefore, we planned to develop a novel nomogram which would help to accurately predict the survival probability of GC patients based on patient and tumor characteristics, laboratory data, and chemotherapy regimens using a nomogram to predict survival probability of GC patients after D2 gastrectomy in a multi-institutional clinical setting with a long-term follow-up. We expected the proposed nomogram to help in objectively and accurately predicting survival probability of GC patients after D2 gastrectomy with adjuvant chemotherapy.

## Materials and methods

### Study population

Patients who had undergone D2 gastrectomy from the First Affiliated Hospital of Nanjing Medical University from January 2008 to August 2012 (cohort 1) and the First Affiliated Hospital of Soochow University from May 2016 to November 2016 (cohort 2) were included in our study. Inclusion criteria: pathologically verified locoregional GC without distant metastasis (Stage I, II, or III, except T1a); at least two cycles of chemotherapy within 2 months after surgery. Exclusion criteria: neoadjuvant treatment; adjuvant radiochemotherapy; gastrointestinal stromal tumor; synchronous malignancies; incomplete clinicopathological data.

In total, 639 GC patients were found to be suitable for the study and they were randomly divided into the training cohort (n = 426) and the validation cohort (n = 213). The median follow-up time was 63.7 (95 CI%: 58.2-67.4) months in all the patients, 63.6 (95 CI%: 57.9-71.7) months in the training cohort, and 62.3 (95 CI%: 54.3-74.3) months in the validation cohort, respectively.

### Data collection

In this study, the evaluated variables included age, gender, tumor location (cardia/fundus, corpus, antrum, or whole), tumor size, depth of tumor invasion, lymph node status, metastatic lymph node ratio, stage, grading, resection margin status, type of gastrectomy (total vs subtotal), platelet, hemoglobin, white blood cell, CEA, CA19-9, and chemotherapy regimens. GC patients were stratified into 2 age groups of younger or older than 60 years of age (14, 15). According to the drugs used in the chemotherapies, the chemotherapy regimens were divided into single chemotherapy regimens (5-FU related drugs), and multiple chemotherapy regimens (paclitaxel-based, oxaliplatin-based therapy, or 5-FU related drugs). The continuous values of CEA level and CA 19-9 level were used for analyses. The seventh edition of the American Joint Committee on Cancer (AJCC) tumor- node- metastasis (TNM) system was used for stage classification. The TNM stage is pathological.

### Statistics

The period of time from the first day of surgery till death or the last date of follow up was used to define OS. Kaplan–Meier curves were used to estimate survival distribution, and the log-rank test was used to evaluate the differences. The Cox proportional hazards model was performed with a 95% CI for the univariate and multivariate analyses to study the risk factors. *P* value ≤ 0.05 was applied to select variables into multivariate analysis. We established a nomogram incorporating the identified potential risk factors to predict survival probability in SPSS software for Windows (version 19; IBM SPSS, Somers, NY, USA) and R Statistical Language (version 2.9; Vienna, Austria). *P* value ≤ 0.05 is the condition for selecting variables into the nomogram after multivariate analysis. The nomogram based on the Cox regression model was used to compute the 1, 3, and 5 year predicted survival probability of GC for each patient. The prognostic performance of a nomogram was measured by the concordance index (c-index), a calibration curve, and area under the receiver operating characteristic (ROC) curve (AUC). Statistical significance was defined as *P <*0.05 in a two-tailed test.

## Results

### Patients

A total of 639 GC patients from the First Affiliated Hospital of Nanjing Medical University and the First Affiliated Hospital of Soochow University were available for our analysis. Demographics for cohort 1 and cohort 2 are presented in **Supplementary Table S1**. The patients were randomly divided into the training cohort (n = 426) and the validation cohort (n = 213). The median age is 58 (range: 18-85) years in the training cohort and 59 (range: 32-85) years in the validation cohort. There were 308 (72.3%) male in the training cohort, and 142 (66.67%) male in the validation cohort. Demographics for training cohort and validation cohort of patients with operable GC are presented in **Table 1**.

**Table 1:** Demographics for training cohort and validation cohort of patients with gastric cancer (N = 639).

Variable	Training cohort (N = 426) N (%)	Validation cohort (N = 213) N (%)	*P* value
Age			
<60 years	231 (54.23)	110 (51.64)	0.537
≥60 years	195 (45.77)	103 (48.36)	
Gender			
Male	308 (72.3)	142 (66.67)	0.141
Female	118 (27.70)	71 (33.33)	
Primary tumor site			
Cardia/fundus	120 (28.17)	51 (23.94)	0.256
Corpus	168 (39.44)	76 (35.68)	
Antrum	127 (29.81)	79 (37.09)	
Whole	11 (2.58)	7 (3.29)	
Depth of tumor invasion			
T1	33 (7.75)	17 (7.98)	0.247
T2	61 (14.32)	21 (9.86)	
T3	117 (27.46)	52 (24.41)	
T4	215 (50.47)	123 (57.75)	
Tumor size			0.333
<6 cm	321 (75.35)	167 (78.4)	
≥6 cm	103 (24.18)	44 (20.66)	
NA	2 (0.47)	2 (0.94)	
Lymph node status			
N0+N1	186 (43.66)	93 (43.66)	1.000
N2+N3	240 (56.34)	120 (56.34)	
Seventh AJCC TNM Stage			
I	54 (12.68)	23 (10.80)	0.771
II	109 (25.59)	54 (25.35)	
III	263 (61.74)	136 (63.85)	
Grading			
Well and Moderately differentiated	91 (21.36)	43 (20.19)	0.731
Poorly differentiated	335 (78.64)	170 (79.81)	
Resection margin			
Negative	322 (75.59)	149 (69.95)	0.127
Positive	104 (24.41)	64 (30.05)	
Type of gastrectomy			
Total	162 (38.03)	77 (36.15)	0.644
Subtotal	264 (61.97)	136 (63.85)	
Hemoglobin g/L [median (IQ values)]	126 (115,141)	126 (115,142)	0.855
White blood cell, ×10^9^/L			
<4	39 (9.15)	21 (9.86)	0.774
≥4	387 (90.85)	192 (90.14)	
Platelet, ×10^9^/L			
<300	379 (88.97)	193 (90.61)	0.523
≥300	47 (11.03)	20 (9.39)	
CEA ng/mL [median (IQ values)]	4 (2, 90)	4 (2, 14)	0.455
CA19-9 U/mL [median (IQ values)]	10 (5, 21)	11 (6, 28)	0.143
Chemotherapy regimens			
Single	25 (5.87)	11 (5.16)	0.716
Multiple	401 (94.13)	202 (94.84)	

NA, not available; metastatic node number: N0, 0; N1, 1-2; N2, 3-6; N3, >6; AJCC, American Joint Committee Cancer; TNM, tumor-node-metastasis; IQ values, interquartile values; CEA, carcinoembryonic antigen; CA19-9, carbohydrate antigen 19-9.

### Univariate and multivariate analyses

Univariate and multivariate analyses were carried out to determine whether the patient’s baseline characteristics, laboratory factors and chemotherapy regimens were significantly associated with survival of GC patients undergoing D2 gastrectomy. The univariate analysis showed that depth of tumor invasion, larger tumor size, positive lymph node status, late TNM stage, positive surgical margin, CEA level, CA19-9 level, and single chemotherapy regimens compared with multiple chemotherapy regimens (**Table 2**) were the factors associated with worse survival.

**Table 2:** Univariate and multivariate analyses of patients with gastric cancer (training cohort, N = 426).

Variable	Univariate analysis			Multivariate analysis		
	HR	95%CI	*P*-value	HR	95%CI	*P*-value
Age ≥60 years vs <60 years	1.24	0.95-1.61	0.113	1.04	0.78-1.40	0.758
Female vs Male	1.07	0.80-1.43	0.638			
Location (corpus/cardia/fundus vs whole)	1.91	0.84-4.30	0.120			
Depth of tumor invasion						
T2 vs T1	1.28	0.52-3.14	0.588			
T3 vs T1	4.16	1.92-9.04	**<0.001**			
T4 vs T1	4.33	2.02-9.27	**<0.001**			
Tumor size (≥6 cm vs <6 cm)	1.60	1.20-2.13	**0.001**	1.23	0.89-1.69	0.208
Lymph node status (positive vs negative)	2.79	2.08-3.75	**<0.001**			
Stage II vs I	2.51	1.22-5.16	**0.013**	1.94	0.89-4.24	0.095
Stage III vs I	6.43	3.28-12.58	**<0.001**	5.11	2.47-10.54	**<0.001**
Grading (poorly vs well and moderately differentiated)	0.92	0.67-1.25	0.586			
Resection margin (positive vs negative)	1.96	1.48-2.61	**<0.001**	1.78	1.31-2.43	**<0.001**
Type of gastrectomy (total vs subtotal)	1.25	0.96-1.64	0.096			
Hemoglobin (<110 g/L vs ≥110 g/L)	1.00	0.99-1.00	0.685			
White blood cell (<4×10^9^/L vs ≥4×10^9^/L)	1.20	0.75-1.92	0.452			
Platelet (≥300×10^9^/L vs <300×10^9^/L)	1.31	0.88-1.97	0.183			
CEA (ng/mL)	1.01	1.00-1.01	**0.022**	1.01	1.00-1.01	**0.024**
CA19-9 (U/mL)	1.00	1.00-1.00	**0.002**	1.00	0.99-1.00	0.247
Chemotherapy regimens (Multiple vs single)	0.49	0.31-0.79	**0.003**	0.48	0.30-0.78	**0.003**

Data in bold indicates *P* < 0.05.

HR, hazard ratio; CI, confidence interval; AJCC, American Joint Committee Cancer; TNM, tumor-node-metastasis; IQ values, interquartile values; CEA, carcinoembryonic antigen; CA19-9, carbohydrate antigen 19-9; LDH, lactic dehydrogenase.

**Figure 1:**
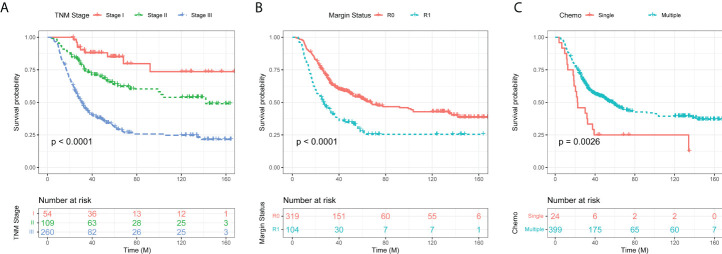
Kaplan–Meier curves of overall survival (OS) in gastric cancer patients according to TNM stage **(A)**, resection margin **(B)**, and chemotherapy regimens **(C)**.

Furthermore, these variables with a *P* value ≤ 0.05 in the univariate analysis were analyzed in a multivariate analysis using a Cox proportional hazards regression model with a forward stepwise procedure. Considering the information regarding the variables of depth of tumor invasion and lymph node status were included in TNM stage, we used TNM stage for the multivariate analysis. Collinearity diagnostics were carried out before the multivariate analysis (**Supplementary Table S2**). The variance inflation factor (VIF) of each variable with statistical significance in the univariate analysis is less than 5, with a mean value of 1.36. Therefore, there is no multi-collinearity among the variable. Late TNM stage, positive resection margin, CEA level, and single chemotherapy regimens compared with multiple chemotherapy regimens with a *P* value ≤ 0.05 were the four factors linked with worse OS on the multivariate analysis. We also used the stepwise method based on AIC criteria to select variables into the nomogram. The results showed that the AIC-value is the smallest when these four variables are selected (**Supplementary Table S3**). All of these findings were validated in the validation cohort. In addition, the survival analyses for these factors using the Kaplan–Meier method are presented in **Figure 1**.

### Nomogram for survival probability

**Figure 2:**
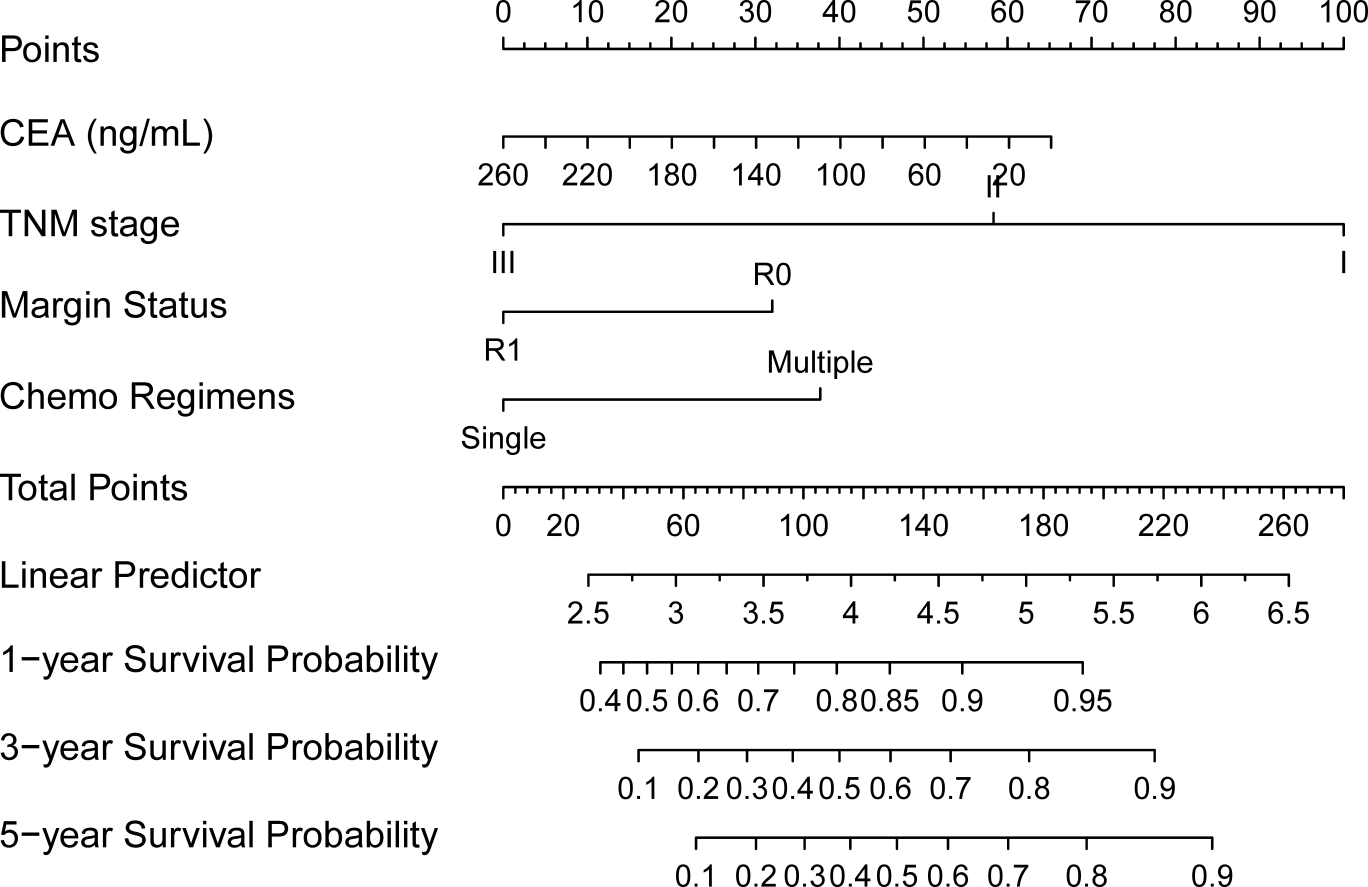
A nomogram for gastric cancer patients undergoing radical surgery and adjuvant chemotherapy. (To use the nomogram, for an individual patient, the value is loaded on each variable axis (the 2nd-5th lines), and a line is drawn upward to determine the number of points received for each variable value (the 1st line). The sum of these numbers is located on the total points axis (the 6th line), and a line is drawn downward to the survival axes (the 8th-10th line, separately) to determine the likelihood of the 1-year, 3-year, and 5-year survival probability.

Next, a nomogram incorporating the four clinical predictors was built up based on the Cox model. We used TNM stage, resection margin status, CEA level, and chemotherapy regimens to establish the nomogram, as shown in **Figure 2**.

For an individual patient, the 1st line shows the number of points received for each variable value which is loaded on each variable axis (the 2nd-5th lines). With the sum of these numbers (the 6th line), we could determine the likelihood of the 1-year, 3-year, and 5-year survival probability showing in the survival axes (the 8th -10th line, separately).

### Validation of predictive accuracy of the nomogram for OS

**Figure 3:**
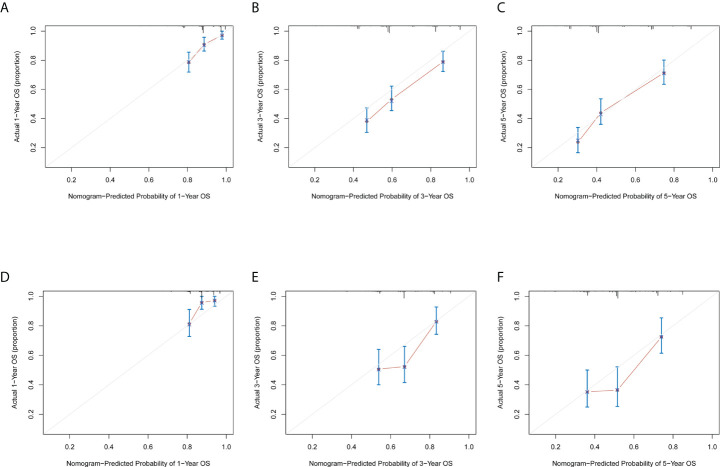
Calibration Curve for training group and validation group. The calibration curve for predicting the 1-year, 3-year, and 5-year survival probability of GC patients in the training cohort **(A–C)** and the validation cohort **(D–F)**. Nomogram-predicted probability of overall survival is plotted on the x-axis; actual overall survival is plotted on the y-axis.

Next, we examined the predictive accuracy and discriminative ability of the nomogram by concordance index (C-index) and calibration curve. For predicting OS, the C-index of the nomogram was 0.676 (95% CI, 0.643–0.709) in the training cohort, and 0.664 (95% CI, 0.609–0.719) in the validation cohort. In addition, the calibration curves in the training cohort (**Figures 3A–C**) and the validation cohort (**Figures 3D–F**) showed good agreement between prediction and observation in the probability of 1-year, 3-year, and 5-year, separately.

**Figure 4:**
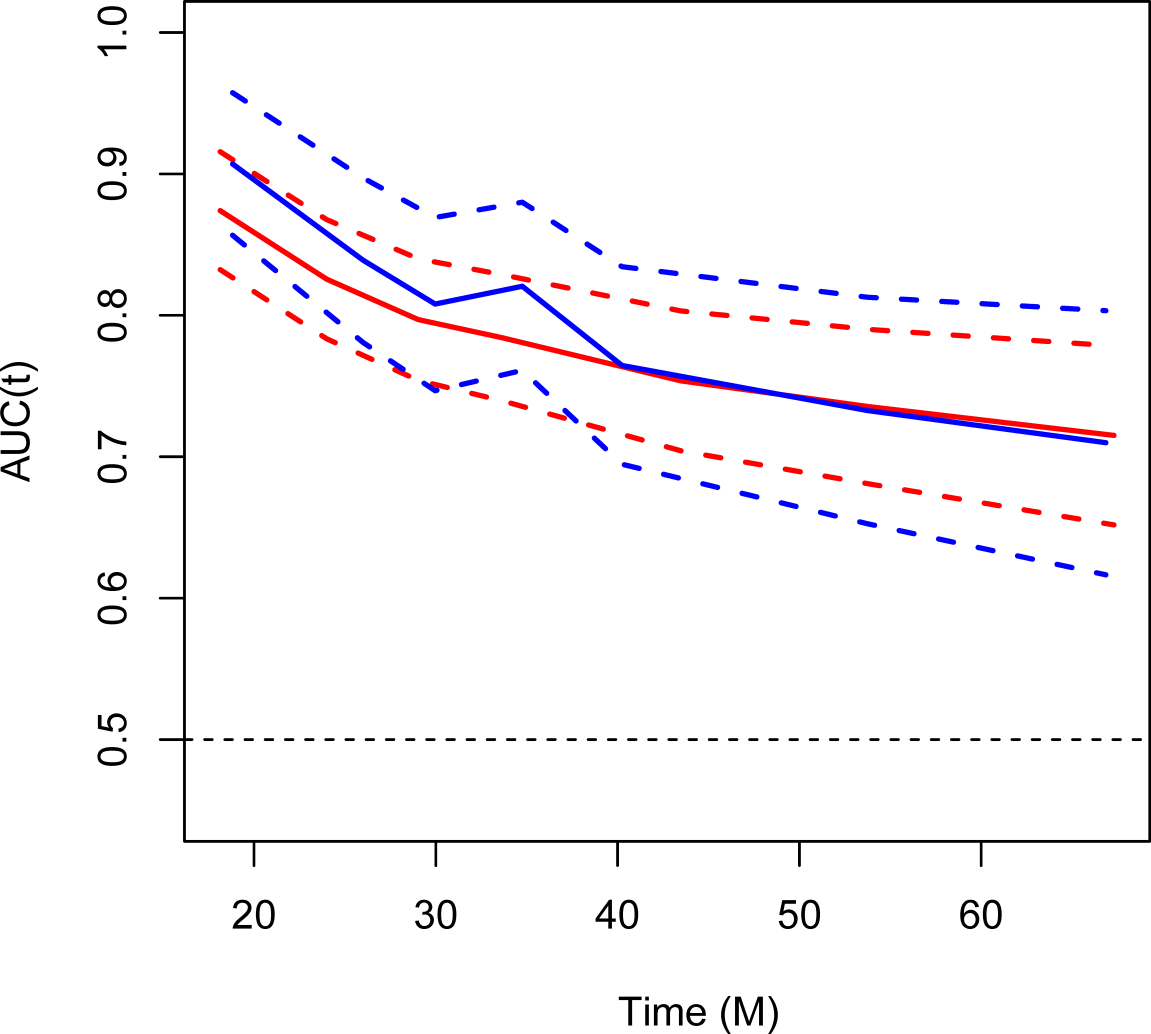
Area under the receiver operating characteristic (ROC) curve (AUC) for assessing predictive accuracy of the nomogram for OS. Red, training cohort; blue, validation cohort.

The AUC values are analyzed by the ROC analysis for assessing predictive accuracy of the nomogram for OS (**Figure 4**). In the training cohort, the AUC values of the ROC projected the 1-year, 3-year, and 5-year OS were 94.43% (95% CI, 90.41%-98.45%), 77.42% (95% CI, 72.83%-82.01%), and 73.03% (95% CI, 67.21%-78.85%), respectively. In the validation cohort, the AUC values of the ROC projected the 1-year, 3-year, and 5-year OS were 96.95% (95% CI, 93.70%-100.00%), 81.54% (95% CI, 75.46%-87.62%), and 73.41% (95% CI, 64.88%-81.94%), respectively. Therefore, the nomogram combined of four predictors, showed powerful prognostic ability in the training cohort and validation cohort.

## Discussion

Gastric cancer (GC) is the third-leading cause of cancer mortality in the world (1). Radical surgery may be the only potential curative treatment for early GC patients. Despite radical surgery, the 5-year survival of GC patients with different stages varies a lot (2, 16). In addition, tumor biomarkers (17–22) and adjunctive therapies (23, 24) are related to the survival time of patients with operable GC. Therefore, further studies are needed to identify a prognostic scoring system and select effective treatments for patients with GC. In this study, we established a nomogram that estimates 1-year, 3-year, and 5-year survival probability of GC patients after D2 gastrectomy combined with adjuvant chemotherapy.

Herein, we included 639 GC patients undergoing D2 gastrectomy and adjuvant chemotherapy from two institutions in China. The patients were randomly divided into the training cohort and the validation cohort. On multivariate analysis, four factors were associated with significantly worse overall survival (OS): late TNM stage, positive resection margin, preoperative CEA level, and single chemotherapy regimens compared with multiple chemotherapy regimens. Furthermore, the four factors were used in the final nomogram for the prediction of 1-year, 3-year, and 5-year survival probability, with accurate calibration and reasonable discrimination (C-index = 0.676 for training cohort, and C-index = 0.664 for validation cohort). In addition, the calibration curve for probability of survival showed good agreement between prediction by nomogram and actual observation.

Some studies have reported different models of nomogram for resectable or advanced GC patients (6, 25, 26). However, these published studies have not established a prognostic nomogram including all the factors concerning patient characteristics, tumor characteristics, preoperative serum markers, and chemotherapy regimens. Kattan et al. developed a postoperative nomogram, which included number of positive/negative lymph nodes resected and depth of invasion to predict 5-year disease-free survival (DFS) after an R0 resection for GC (6). Muneoka et al. reported a nomogram for 5-year relapse-free survival (RFS) of advanced GC patients who had undergone curative resection for stage II or III GC and never received any adjuvant chemotherapy (8).The study of Muneoka et al. revealed that depth of invasion and number of metastasized lymph nodes were significant prognostic factors affecting RFS. In our present study, we established a nomogram that estimates 1-year, 3-year, and 5-year survival probability of GC patients after D2 gastrectomy combined with adjuvant chemotherapy. In accordance with the reported studies, we also found that depth of tumor invasion, positive lymph node status, higher metastatic lymph node ratio, late TNM stage were linked with worse survival in univariate Cox analysis. In addition, the late TNM stage retained a significant prognostic factor associated with worse OS of GC patients undergoing D2 gastrectomy combined with chemotherapy in multivariate analysis. Moreover, the TNM stage is considered as one of the predictors incorporated in our proposed nomogram for operable GC patients undergoing chemotherapy treatment. Kattan and Muneoka et al. focused on studying the prognostic factors of patient and tumor characteristics. However, in our study, along with patient and tumor characteristics, we also took preoperative serum markers and chemotherapy regimens into consideration.

Many large trials have demonstrated that a perioperative chemotherapy and postoperative chemotherapy regimen significantly improved PFS or OS in patients with resectable GC (27–30). It was found that the OS and RFS were improved among those Asian patients who had received postoperative adjuvant therapy with S-1 (an oral Fluoropyrimidine) after a D2 dissection for locally advanced GC (29, 31). The CLASSIC trial showed that adjuvant capecitabine plus oxaliplatin significantly improved 3-year DFS, 5-year DFS, and 5-year OS after D2 gastrectomy for patients with stage II or III GC, compared with observation (32, 33). Kawamoto et al. reported the SNOW regimen (a combination of S-1, nab-paclitaxel and oxaliplatin) as a promising new triplet therapy for advanced GC (34). Han et al. established a nomogram for GC patients after D2 gastrectomy to predict long-term survival (7). In Han’s study, adjuvant chemotherapy failed to demonstrate significance in the Cox regression model and was excluded from the nomogram. Han et al. claimed that adjuvant chemotherapy was not a significant variable in their study because of two main reasons: a) no standard regimens had been established after gastrectomy with D2 lymphadenectomy before 2007; b) in their institution, adjuvant chemotherapy based on fluorouracil and platinum was omitted only in patients with stage II or III GC with poor functional status and reluctant to receive chemotherapy. In our study, all the patients were treated with standard D2 gastrectomy after January 2008 and followed by adjuvant chemotherapy. We compared the treatment effects among single chemotherapy regimens (5-FU related drugs), and multiple chemotherapy regimens (paclitaxel, oxaliplatin, or 5-FU related drugs). Our results showed that multiple chemotherapy regimens were associated with significantly better OS as compared with single chemotherapy regimens on univariate and multivariate analyses. In addition, chemotherapy regimens were used in the nomogram to predict 1-year, 3-year, and 5-year OS of GC patients undergoing D2 gastrectomy combined with adjuvant chemotherapy.

Serum markers have been considered as significant diagnostic and prognostic factors for cancer patients (7, 35, 36). Duraker et al. documented that OS was significantly poorer in CEA positive patients (log-rank test, *P* = 0.003) (35). In a prospective study, Takahashi et al. depicted the role of serum CEA level in monitoring GC recurrence postoperatively, especially in patients with high preoperative levels (36). Our study also demonstrated elevated CEA level as a prognostic indicator for poorer OS in GC patients undergoing radical surgery and chemotherapy.

As no standard targeted therapy regimens had been established in 2008, we did not include the targeted therapy regimens. With the success of phase 3 trials like ToGA (37), REGARD (38), RAINBOW (39) and others in advanced GC patients, the targeted therapies have to be taken into investigation in future studies. A limitation of this study is a retrospective study at two institutions. Therefore, the proposed nomogram still needs to be examined to objectively and accurately predict survival probability of GC patients in a prospective multi-institutional clinical setting.

In conclusion, we established a nomogram for the prediction of 1-year, 3-year, and 5-year survival probability of GC patients after D2 gastrectomy combined with adjuvant chemotherapy. The nomogram incorporates TNM stage, resection margin, preoperative CEA level, and chemotherapy regimens based on the Cox model with accurate calibration and reasonable discrimination. These findings might shed light on prospective multi-institutional trials in operable GC toward clinical applications of the proposed nomogram.

## Data availability statement

The original contributions presented in the study are included in the article/**Supplementary Material**. Further inquiries can be directed to the corresponding authors.

## Ethics statement

The studies involving human participants were reviewed and approved by the Ethics Committee of The First Affiliated Hospital of Nanjing Medical University. The patients/participants provided their written informed consent to participate in this study. Written informed consent was obtained from the individual(s) for the publication of any potentially identifiable images or data included in this article.

## Author contributions

LM and GC contributed equally to this work. WL and XC conceived the study and designed the outline of the research. LM, GC, DW, and KZ performed the study, analyzed the data, and drafted the manuscript. FZ, JT, JZ, SH, and DL contributed to the data collection and follow-up. YG, MT, and YS provided clinical guidance. OR helped in designing and revising the manuscript. All authors read and approved the final manuscript.

## Funding

This study was partly supported by the National Natural Science Foundation of China (No. 82102981), the open research fund of Jiangsu Key Laboratory for Design and Manufacture of Micro-Nano Biomedical Instruments, Southeast University (No. KF202103), and Beijing Science and Technology Innovation Medical Development Foundation (KC2021-JX-0186-124).

## Acknowledgments

We would like to thank the Core Facility of the First Affiliated Hospital of Nanjing Medical University for the help in this work.

## Conflict of interest

The authors declare that the research was conducted in the absence of any commercial or financial relationships that could be construed as a potential conflict of interest.

The handling editor JW declared a shared parent affiliation with the authors LM, GC, KZ, FZ, JZ, YG, YS, and XC at the time of review

## Publisher’s note

All claims expressed in this article are solely those of the authors and do not necessarily represent those of their affiliated organizations, or those of the publisher, the editors and the reviewers. Any product that may be evaluated in this article, or claim that may be made by its manufacturer, is not guaranteed or endorsed by the publisher.
