# Supplementary material for: A nomogram to predict survival probability of gastric cancer patients undergoing radical surgery and adjuvant chemotherapy
Source: Front Oncol. 2022 Aug 5;12:893998. doi: 10.3389/fonc.2022.893998 (PMC9389342; doi:10.3389/fonc.2022.893998)
Supplement: Supplementary file 1 [file DataSheet_1.docx]

| Supplementary Table 1. Demographics for cohort 1 and cohort 2 of patients with gastric cancer (N = 639) | | | |
| --- | --- | --- | --- |
| Variable | cohort 1 (N = 579) N (%) | cohort 2 (N = 60) N (%) | *P* |
| Age |  |  | 0.412 |
| <60 years | 312 (53.89) | 29 (48.33) |  |
| ≥60 years | 267 (46.11) | 31 (51.67) |  |
| Gender |  |  | 0.414 |
| Male | 405 (69.95) | 45 (75) |  |
| Female | 174 (30.05) | 15 (25) |  |
| Primary tumor site |  |  | 0.476 |
| Cardia/fundus | 153 (26.42) | 18 (30) |  |
| Corpus | 226 (39.03) | 18 (30) |  |
| Antrum | 183 (31.61) | 23 (38.33) |  |
| Whole | 17 (2.94) | 1 (1.67) |  |
| Depth of tumor invasion |  |  | **<0.001** |
| T1 | 49 (8.46) | 1 (1.67) |  |
| T2 | 80 (13.82) | 2 (3.33) |  |
| T3 | 118 (20.38) | 51 (85) |  |
| T4 | 332 (57.34) | 6 (10) |  |
| Tumor size |  |  | 0.731 |
| <6 cm | 446 (77.03) | 42 (70.00) |  |
| ≥6 cm | 133 (22.97) | 14 (23.33) |  |
| NA | 0 (0.00) | 4 (6.67) |  |
| Lymph node status |  |  | **0.001** |
| N0+N1 | 265 (45.77) | 14 (23.33) |  |
| N2+N3 | 314 (54.23) | 46 (76.67) |  |
| Seventh AJCC TNM Stage |  |  | **0.021** |
| I | 76 (13.13) | 1 (1.67) |  |
| II | 149 (25.73) | 14 (23.33) |  |
| III | 354 (61.14) | 45 (75) |  |
| Grading |  |  | **<0.001** |
| Well and Moderately differentiated | 88 (15.2) | 46 (76.67) |  |
| Poorly differentiated | 491 (84.8) | 14 (23.33) |  |
| Resection margin |  |  | **<0.001** |
| Negative | 414 (71.50) | 57 (95.00) |  |
| Positive | 165 (28.50) | 3 (5.00) |  |
| Type of gastrectomy |  |  | **0.037** |
| Total | 224 (38.69) | 15 (25.00) |  |
| Subtotal | 335 (61.31) | 45 (75.00) |  |
| Hemoglobin g/L [median (IQ values)] | 125 (115,142) | 130 (115,145) | 0.396 |
| White blood cell, ×10^9^/L |  |  | 0.118 |
| <4 | 51 (8.81) | 9 (15.00) |  |
| ≥4 | 528 (91.19) | 51 (85.00) |  |
| Platelet, ×10^9^/L |  |  | 0.754 |
| <300 | 519 (89.64) | 53 (88.33) |  |
| ≥300 | 60 (10.36) | 7 (11.67) |  |
| CEA ng/mL [median (IQ values)] | 4.0 (2.0, 12.0) | 1.5 (0.9,2.5) | **<0.001** |
| CA19-9 U/mL [median (IQ values)] | 10.0 (5.0, 22.0) | 15.2 (6.2, 124.4) | 0.229 |
| Chemotherapy regimens |  |  | 0.239* |
| Single | 35 (6.04) | 1 (1.67) |  |
| Multiple | 544 (93.96) | 59 (98.33) |  |

Note: Data in bold indicates *P* < 0.05; *Fisher’s exact test.

Abbreviations: NA, not available; metastatic node number: N0, 0; N1, 1-2; N2, 3-6; N3, >6; AJCC, American Joint Committee Cancer; TNM, tumor-node-metastasis; IQ values, interquartile values; CEA, carcinoembryonic antigen; CA19-9, carbohydrate antigen 19-9.

Supplementary Table 2. Collinearity diagnostics

| Variable | VIF |
| --- | --- |
| TNM Stage | 2.26 |
| Resection margin | 1.06 |
| Chemotherapy regimens | 1.02 |
| CEA level | 1.01 |
| CA19-9 level | 1.02 |
| Tumor size | 1.07 |
| Age | 1.03 |
| Mean | 1.36 |

Abbreviations: VIF, Variance Inflation Factor; TNM, tumor-node-metastasis;

CEA, carcinoembryonic antigen; CA19-9, carbohydrate antigen 19-9.

Supplementary Table 3. AIC criteria of selecting variables into the nomogram

| Selected variables | AIC-value |
| --- | --- |
| TNM Stage | 2015.06 |
| TNM Stage + Resection margin | 2002.48 |
| TNM Stage + Resection margin + Chemotherapy regimens | 1996.59 |
| TNM Stage + Resection margin + Chemotherapy regimens + CEA level | 1994.47 |

Abbreviations: TNM, tumor-node-metastasis; CEA, carcinoembryonic antigen.
